# A New Incision for Appendectomy

**Published:** 1919-03

**Authors:** Leigh F. Watson

**Affiliations:** 30 N. Michigan Avenue; Chicago


### A New Incision for Appendectomy.

LEIGH F. WATSON, M. D., CHICAGO.

Many writers have noted that in the cadaver the base of the appendix is found at McBurney's point, while in the living subject it is below this point, usually on a level with the center of Poupart's ligament. A number of operators have called attention to the ease with which the appendix can be removed when operating for right inguinal hernia. Since 1910, I have used a new incision, with its center over the base of the appendix, and believe that in many cases it is an improvement over those in general use.

Incision: A point one and one-half inches from the right anterior superior spine, on a level with a line connecting the two superior spines, is selected for the beginning of a vertical incision which extends directly downward for two to three inches to a point just above, and to the inner side of the internal abdominal ring.

Advantages: Traction to expose the appendix is avoided, because this incision, in the external oblique and its aponeurosis, the most resistant structures, is directly over the base of the appendix. It can be enlarged without weakening the abdominal wall. The ilio-hypogastric and ilio-inguinal nerves are not injured because the incision lies between them. Because this incision is made over the cecum, the small intestines do not crowd into the wound as they do when the McBurney and lateral rectus incisions are used.

30 N. Michigan Avenue.

